# RGB Pixel Brightness Characteristics of Linked Color Imaging in Early Gastric Cancer: A Pilot Study

**DOI:** 10.1155/2020/2105874

**Published:** 2020-03-31

**Authors:** Xue Sun, Li Zhao

**Affiliations:** ^1^VIP Department and General Medicine Department, Beijing Hospital, National Center of Gerontology, Institute of Geriatric Medicine, Chinese Academy of Medical Sciences, China 100730; ^2^Department of Gastroenterology, Beijing Hospital, National Center of Gerontology, Institute of Geriatric Medicine, Chinese Academy of Medical Sciences, China 100730

## Abstract

**Background and Aims:**

Linked color imaging (LCI) helps screen and diagnose for early gastric cancer by color contrast in different mucosa. RGB (red, green, and blue) pixel brightness quantifies colors, which is relatively objective. Limited studies have combined LCI images with RGB to help screen for early gastric cancer (EGC). We aimed to evaluate the RGB pixel brightness characteristics of EGC and noncancer areas in LCI images.

**Methods:**

We retrospectively reviewed early gastric cancer (EGC) patients and LCI images. All pictures were evaluated by at least two endoscopic physicians. RGB pixel brightness analysis of LCI images was performed in MATLAB software to compare the cancer with noncancer areas. Receiver operating characteristic (ROC) curve was analyzed for sensitivity, specificity, cut-off, and area under the curve (AUC).

**Results:**

Overall, 38 early gastric cancer patients were enrolled with 38 LCI images. Pixel brightness of red, green, and blue in cancer was remarkably higher than those in noncancer areas (190.24 ± 37.10 vs. 160.00 ± 40.35, *p* < 0.001; 117.96 ± 33.91 vs. 105.33 ± 30.01, *p* = 0.039; 114.36 ± 34.88 vs. 90.93 ± 30.14, *p* < 0.001, respectively). *Helicobacter plyori* (Hp) infection was not relevant to RGB distribution of EGC. Whether the score of Kyoto Classification of Gastritis (KCG) is ≥4 or <4, the pixel brightness of red, green, and blue was not disturbed in both cancer and noncancer (*p* > 0.05). Receiver operating characteristic (ROC) curve for differentiating cancer from noncancer was calculated. The maximum area under the curve (AUC) was 0.767 in B/G, with a sensitivity of 0.605, a specificity of 0.921, and a cut-off of 0.97.

**Conclusions:**

RGB pixel brightness was useful and more objective in distinguishing early gastric cancer for LCI images.

## 1. Background and Aims

China is a country with a high incidence of gastric cancer. According to the latest World Health Organization data, the estimated age-standardized incidence rate of gastric cancer in 2018 is 20.7 per 100,000 [1]. It is also the third-leading cause of cancer-related death. Symptoms of EGC are concealed. Most of the patients are diagnosed at an advanced stage with a poor five-year survival rate. Thus, screening and early diagnosis for EGC is vital, especially to those with high risk factors, such as *Helicobacter pylori* infection [2] and intestinal metaplasia. White-light endoscopic observation alone is not enough. Blue laser imaging (BLI) is a newly developed image-enhanced endoscopy (IEE) system, which has two laser light sources. The LCI observation mode is one of the four modes offered by the IEE system. The main feature is color enhancement that makes it easier to recognize the slight difference in mucosal color [3]. For instance, lesions with HP infection were identified with a diffusion of red color [4]. Lesions of intestinal metaplasia were in purple or lavender color sign [5]. EGC in LCI were usually presented as a red area with yellow color, while those of advanced cancer were presented as a red area with white color [6]. Taking into account physicians' subjective judgment, missed diagnosis is unavoidable [7], especially for inexperienced endoscopic physicians.

RGB (red, green, and blue) color is an optical tricolor. Different proportions of RGB superposition formed different colors on the monitor. Considering that colors can be quantified, the RGB pixel brightness model may be relatively objective for EGC screening. Based on another pilot study, RGB pixel brightness is different between LCI and white-light imaging (WLI) in gastritis patients [8]. To the best of our knowledge, whether or not the KCG score and Hp infection would affect the judgment of RGB pixel brightness for early gastric cancer was unknown. In this study, we aimed at the RGB pixel brightness characteristics of cancer and noncancer areas in EGC patients in LCI images, also considering the effect of KGC and Hp infection on the RGB distribution.

## 2. Methods

This is a single-center retrospective study. We analyzed WLI and LCI images from patients with early gastric cancer diagnosed between March 2018 and August 2019. Inclusion criteria are as follows: (1) The patient underwent both WLI and LCI. (2) The patient was firstly diagnosed with early gastric cancer and confirmed by pathology. (3) There was a negative surgical margin. (4) The cancer area and the adjacent noncancer area with the same size and similar brightness could be found in the same LCI image. Patients who have advanced gastric cancer, who were Hp negative after eradication therapy, or those who did not meet the criteria listed were excluded.

We numbered the included patients firstly and collected basic information, including gender, age, HP infection, and family history of gastric cancer. All images were acquired from the LASEREO system (VP4450HD; Fujifilm Corporation, Tokyo, Japan). We assessed five endoscopic findings included in the Kyoto Classification of Gastritis (KCG) independently [9]: (1) atrophy—none (no atrophy), mild (C1, C2), moderate (C3, O1), and severe (O2, O3); (2) intestinal metaplasia—greyish-white, slightly opalescent, flat, elevated lesion of various sizes; (3) enlarged folds—enlarged and tortuous gastric body folds, not flattening upon insufflation; (4) nodularity—nodular or granular elevated lesions measuring 2-3 mm are uniformly distributed in the antrum and angle; and (5) diffuse redness. KCG was evaluated by at least two experienced endoscopic physicians. If the two endoscopic physicians had different opinions, then they sought the advice of a third senior physician.

All lesions were endoscopically resected by endoscopic submucosal dissection (ESD). The sizes and margins of cancer were evaluated in resected specimens to make sure the adjacent mucosa was benign as possible. Based on the pathological findings of EGC, we considered the adjacent mucosae as the noncancer area. Then, we evaluated the RGB contrast between cancer and the surrounding noncancer mucosa in the same view without magnification. For each patient, we selected one clear LCI image in close-up or middle view. The images in which the cancer area and the adjacent noncancer area with the same size and similar brightness could not be found at the same time were excluded.

**Figure 1:**
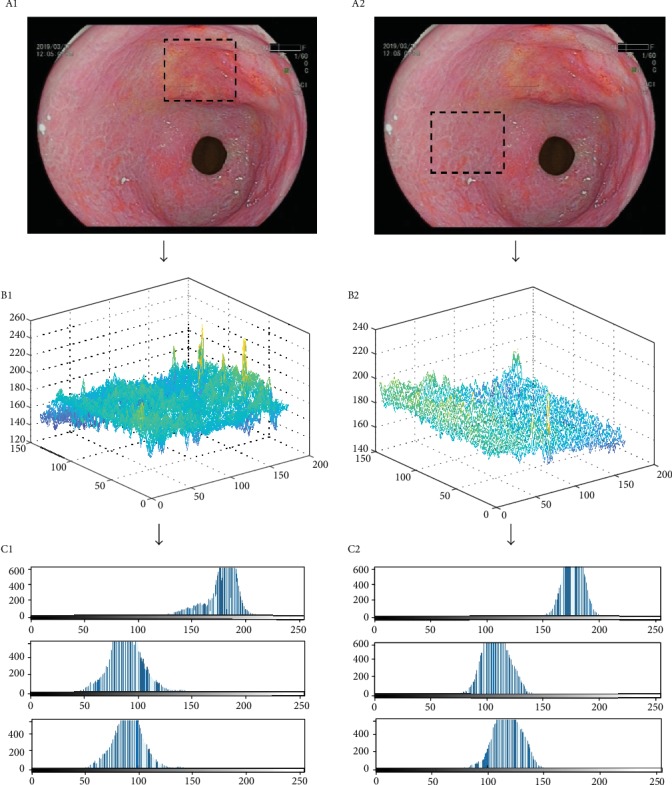
The selected regions of cancer (a1) and noncancer (a2) areas, with the same size and similar brightness. The three-dimensional graphs of RGB basic analysis in cancer (b1) and noncancer (b2) areas and RGB pixel brightness distribution of cancer (c1) and noncancer (c2) areas; the first row is red, the second row is green, and the third row is blue.

MATLAB software (MATLAB_R2017b for Mac) was used to calculate pixel brightness of LCI images (Figure 1). In MATLAB software, we transformed images into double precision data that were displayed as three-dimensional graphs. Then, the software would analyze red, green, and blue distribution and form three two-dimensional matrices. Each RGB value was extracted for further statistics. This study was registered in the Chinese Clinical Trial Registry (ChiCTR1900021827) in March 11th, 2019. We have obtained ethical approval from the Institutional Review Board of the Beijing Hospital, National Center of Gerontology (No. 2019BJYYEC-023-01).

### 2.1. Statistics

The statistical analyses were conducted in SPSS version 25.0 for Mac. Image quantization analysis was performed in MATLAB software. Continuous variables with normal distribution were presented as the means ± standard deviation and compared using Student's *t*-test. Nonnormal distribution variables were presented as median and quartile. A paired-sample *t*-test was used in comparing the pixel brightness of red, green, and blue between the cancer and noncancer areas. ROC curves of a different RGB arithmetic were analyzed. The sensitivity, specificity, cut-off, and AUC were calculated, respectively. A *p* value < 0.05 in each analysis was considered to be statistically significant.

## 3. Results

### 3.1. Clinical Characteristics

**Figure 2:**
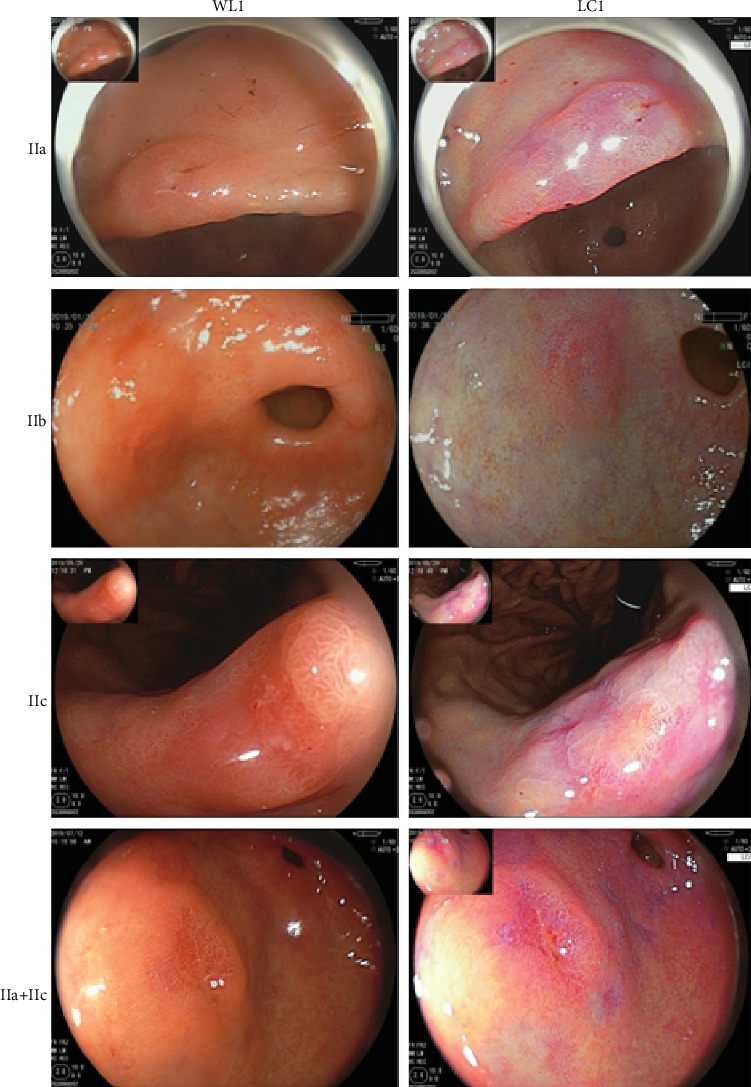
Different shapes of EGC include IIa, IIb, IIc, and IIa+IIc. Each row indicates the same EGC in WLI and LCI separately.

38 patients diagnosed with early gastric cancer and 38 images were enrolled. The patient characteristics in this study are shown in Table 1. Different shapes of EGC were demonstrated in Figure 2. Patients negative for Hp infection referred to those uninfected for Hp previously and negative in recent findings. Pathological diagnosis in 36 patients was well differentiated. Two patients were characterized as poorly differentiated. One of the lesions covered both lessor curvature and anterior wall of gastric antrum. All lesions diagnosed as EGC were limited to the mucosa or submucosa after endoscopic resection. The adjacent mucosa histological finding of EGC were examined using resected specimens of cancer.

### 3.2. RGB Pixel Brightness in Cancer and Noncancer

In order to ensure that the RGB results are not disturbed by different brightness characteristics caused by different distances from the light source, we selected the cancer area and the noncancer area with the same size and similar brightness in the same LCI image. The pixel brightness of red, green, and blue in the cancer area was all significantly higher than that in the noncancer area (Table 2). Whether HP infection was positive or negative, there was no statistical difference in both the cancer and noncancer areas in RGB distribution (Table 3.).

### 3.3. KCG

According to the KCG score based on WLI, LCI images were classified into two groups, KCG ≥ 4 and KCG < 4. Whether KCG ≥ 4 or KCG < 4, the red and blue pixel brightness in cancer was statistically higher than that in noncancer. Nevertheless, the green pixel brightness was higher in cancer in both KCG ≥ 4 and KCG < 4, but without statistical significance. There is no difference of RGB pixel brightness between KCG ≥ 4 and KCG < 4 (Table 4).

### 3.4. ROC

**Figure 3:**
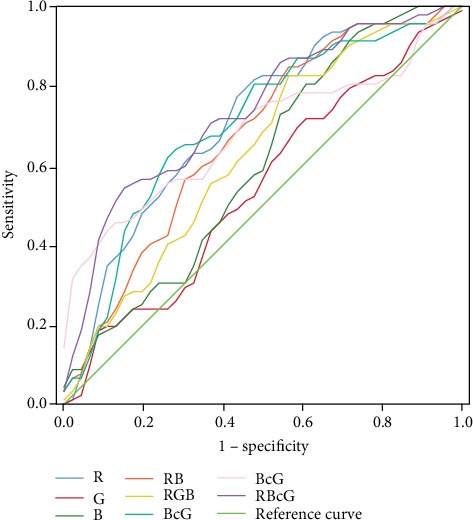
Comparison of ROC curves for different colors and their algorithms for screening early gastric cancer.

On the grounds of the data above, we created ROC curves for differentiating cancer from noncancer by different pixel brightness characteristics and various calculation methods (Table 5, Figure 3; ROC). The maximum AUC was 0.767 in B/G, with a sensitivity of 0.605, a specificity of 0.921, and a cut-off of 0.97. R/G had the highest sensitivity of 0.816. Thus, R/G and B/G may be potential markers for EGC screening during endoscopy.

## 4. Discussion

In the present study, we used a quantitative approach to evaluate the color difference between a malignant lesion and the surrounding mucosa. Our study demonstrated the feasibility of RGB pixel brightness to distinguish EGC from noncancer areas, especially in R and B. Those three-dimensional graphs transformed from LCI images provided a more intuitive method to analyze EGC colors. Various calculation methods in ROC provided the highest sensitivity in R/G, the highest specificity in B/G, and the maximum AUC in B/G. These calculation methods may be potential endoscopic makers for EGC.

RGB analysis is defined by the International Electrotechnical Commission as being able to describe the three color channels and their mutual superposition, which has been widely used in image processing and digital media. Sun et al. firstly applied the ratio of RGB in LCI images to diagnose gastric mucosal lesions, and they discovered that R/(G+B) was the maximum AUC with a sensitivity of 0.514 and a specificity of 0.773 [8]. However, most cases were chronic gastritis and only 2 cases were gastric cancer. In the present study, we focused on EGC and conducted different calculation methods including R/(G+B). Considering the differences between cancer and noncancer in different elements, we finally confirmed B/G as the potential marker. These values with high sensitivity and specificity can act as an evaluation index to be applied in clinical practice for the discrimination between EGC and benign lesions. Moreover, we will seek more accurate calculation methods. Regarding quantification of endoscopic findings related to EGC, other color component values (L∗, a∗, and b∗) [10] were used to confirm that LCI images have a higher color contrast between EGC and surrounding mucosa compared to WLI. In addition, the L∗a∗b∗ color space may even have an association with surface blood vessel density in EGC lesions [11].

Our study chose the RGB model instead of the L∗a∗b∗ color model. The main reason is that L∗ represents luminosity, which is easily inconsistent under endoscopy and may affect assessment. Besides, L∗a∗b∗ is regarded as a device-independent color model. L∗a∗b∗ describes the way colors are displayed, not the amount of specific colors required by devices [12]. Even though RGB and L∗a∗b∗ could convert to each other sometimes [13], the RGB model already contains enough colors on the screen.

Furthermore, the RGB color space demonstrates device-related color, which means that RGB reflects the original color of the gastroscope display. For this reason, prior computation of RGB and other statistical measures are necessary in deep convolutional neural networks for automatic classification of gastric carcinoma [14]. In another machine-learning study, extraction of RGB differences helped prove that acetic acid-indigo carmine chromoendoscopy was suitable for the diagnosis of EGC [15]. RGB analysis plays an important role in the diagnosis of not only EGC but also colorectal lesions. For instance, RGB and their transformation values assisted in forming a computer-aided diagnostic system, which is based on LCI images to predict the histological results of colorectal adenomatous polyps [16]. In short, RGB combined with LCI images is an objective and quantitative method for screening EGC.

Compared to WLI, LCI particularly enhanced the visibility of a normochromic, flat, and depressed lesion, which contributes to the early detection of gastric mucosal cancer [3]. Therefore, RGB pixel brightness as a potential way to improve the accuracy of EGC screening is an additional benefit to LCI. KCG was proposed at the 85th Congress of the Japan Gastroenterological Endoscopy Society [17]. Endoscopic detection of atrophy, intestinal metaplasia, and diffuse redness were associated with the risk of gastric cancer [9]. In this study, the KCG score did not affect RGB from distinguishing cancer from noncancer. Red and blue pixel brightness was still higher in cancer than in noncancer. Nevertheless, RGB pixel brightness did not reflect much difference between KCG score ≥ 4 and <4. Although a higher KCG score refers to a higher risk of gastric cancer, fortunately, the RGB characteristics of EGC were not interfered by KCG. Due to our small sample size, we only set a KCG score of 4 as the cutoff value. We will continue to explore the effect of KCG on RGB distribution. Maybe, there would be a slight difference in noncancer when expanding our sample size.

Benefiting from LCI-enhancing endoscopic images of the diffuse redness of the fundic gland mucosa, LCI is more useful for the diagnosis of HP infection than WLI [4]. LCI could play a valuable initial screening method for real-time diagnosis of HP infection with a high accuracy [18]. After successful Hp eradication, some LCI features like map-like redness and the absence of the regular arrangement of collecting venules relate to gastric cancer [19]. Nevertheless, HP infection has no influence on RGB diagnosis of EGC in our study. Whether the patient has HP infection or not, the RGB distribution of EGC differs from the surrounding mucosa. Another study with Lab mode revealed that LCI allowed clear endoscopic visualization of the atrophic border, whether it has an HP-infected or HP-eradicated status [20]. This result is similar with our study.

In the meantime, the 3-dimensional surface plots that were automatically generated by RGB pixel brightness were significantly different between the malignant lesion and the surrounding mucosa. Thus, they may also be used as important references to identify the EGC because of its visualization.

Furthermore, Sun et al. [21] established an LCI-based endoscopic model called color-microstructure-vessel criteria to extract color features, which allowed for predicting chronic nonatrophic gastritis, chronic atrophic gastritis, and gastric cancer. This is a creative idea and could be combined with RGB to improve the accuracy and efficiency of diagnosing gastric mucosal lesions and benefit target biopsy. In addition, RGB may assist in deep learning in artificial intelligence to cultivate an excellent ability to diagnose EGC.

Our study also has several limitations. Firstly, this pilot study was a single-center retrospective analysis and stored images were retrieved from our electronic database. There remains the possibility of selection bias. Therefore, real-time diagnostic assessments will be performed in the future. Secondly, the sample size was small and only 38 LCI images were enrolled, while EGC has multifarious types and colors. In addition, the demarcation points (KCG ≥ 4 and KCG < 4) may not be suitable and a large sample size and stratified analysis are needed. However, as a pilot study, our results could be able to provide relatively reliable evidence for the clinical usage of computer-aided RGB-based analysis of LCI images in the differentiation of EGC. Thirdly, we excluded Hp-negative patients after eradication therapy. We do not know whether those patients' RGB characteristics of EGC would change. A multicenter large-scale prospective investigation on the clinical application of computer-aided fractal-based analysis of LCI images in the differentiation of EGC is expected.

In summary, LCI is a new revolution in endoscopic diagnostic technology, which significantly improves the rate of EGC discovery by the stronger color difference between lesion and background mucosa. We demonstrated that the RGB color mode could quantify the LCI image difference between the EGC areas and the surrounding areas, without being affected by KCG or Hp infection. This quantitative approach may allow easy recognition and early detection of EGC for either experienced or inexperienced endoscopic physicians during endoscopic procedures. Furthermore, it is also possible to embed the prior knowledge of RGB characteristics into a machine-learning model to pursue the validity and accuracy for screening EGC.

## Figures and Tables

**Table 1:** Patients' main clinical characteristics.

	Patient (*n* = 38)
Age (years)	67.41 ± 10.45
Gender (male/female)	27/11
Shape of lesion	
IIa	10 (26.32%)
IIb	2 (5.26%)
IIc	4 (10.53%)
IIa+IIc	22 (57.89%)
Location of lesion (longitudinal)	
Upper third	6 (15.79%)
Middle third	9 (23.68%)
Lower third	23 (60.53%)
Location of lesion (circumferential)	
Anterior wall	7 (18.24%)
Posterior wall	11 (28.95%)
Greater curvature	8 (21.05%)
Lessor curvature	12 (31.58%)
Family history of gastric cancer	3 (7.89%)
HP infection	
Positive	18 (47.37%)
Negative	20 (52.63%)

**Table 2:** Paired-sample *t* test for RGB pixel brightness in the cancer area and the noncancer area.

	Red	Green	Blue
Cancer area	190.24 ± 37.10	117.96 ± 33.91	114.36 ± 34.88
Noncancer area	160.00 ± 40.35	105.33 ± 30.01	90.93 ± 30.14
*t*	5.396	2.143	4.495
*P* value	<0.001	0.039	<0.001

**Table 3:** *t* test for cancer and noncancer areas whether positive or negative for HP infection.

	Number	Red		Green		Blue	
		Cancer	Noncancer	Cancer	Noncancer	Cancer	Noncancer
HP+	18	181.90 ± 39.65	153.99 ± 42.18	115.10 ± 32.64	102.08 ± 34.42	115.20 ± 33.78	89.28 ± 32.30
HP-	19	200.12 ± 33.61	164.84 ± 39.39	122.30 ± 36.21	108.25 ± 26.08	114.67 ± 37.58	92.65 ± 29.45
*t*		-1.503	-0.807	-0.636	-0.612	0.045	-0.330
*P* value		0.142	0.425	0.529	0.544	0.964	0.743

**Table 4:** *t* test for cancer and noncancer areas in red, green, and blue in KCG ≥ 4 and KCG < 4 separately.

KCG	*n*	Red			Green			Blue		
		Cancer	Noncancer	*p*	Cancer	Noncancer	*p*	Cancer	Noncancer	*p*
≥4	22	189.34 ± 40.54	158.70 ± 42.90	0.002	117.44 ± 39.11	106.86 ± 35.21	0.246	112.14 ± 39.02	92.35 ± 36.38	0.021
<4	17	191.36 ± 35.57	161.70 ± 38.21	0.001	118.61 ± 27.31	103.44 ± 22.73	0.063	117.10 ± 29.93	89.18 ± 21.01	0.001
*P* value		0.870	0.829		0.917	0.732		0.669	0.752	

KCG: Kyoto Classification of Gastritis score.

**Table 5:** Comparison of ROC for different colors and their algorithms, with sensitivity, specificity, cut-off, and AUC.

Curve	R	G	B	R+B	R+B+G	R/G	B/G	(R+B)/G
Sensitivity	0.737	0.316	0.579	0.500	0.632	0.816	0.605	0.711
Specificity	0.632	0.895	0.737	0.895	0.711	0.447	0.921	0.711
Cut-off	178.76	136.21	112.87	310.52	394.31	1.45	0.97	2.44
AUC	0.721	0.607	0.690	0.722	0.689	0.649	0.767	0.745

## Data Availability

The data used to support the findings of this study are available from the corresponding author upon request.
